# Investigating association of perineural invasion on prostate biopsy with Gleason score upgrading at prostatectomy: A multi‐institutional analysis

**DOI:** 10.1002/cam4.2920

**Published:** 2020-03-18

**Authors:** Andrew R. Barsky, Ryan D. Kraus, Ruben Carmona, Patricia M. G. Santos, Carrie Li, Lauren E. Schwartz, Leslie K. Ballas, Neha Vapiwala

**Affiliations:** ^1^ Department of Radiation Oncology Perelman School of Medicine of the University of Pennsylvania Philadelphia PA USA; ^2^ Department of Radiation Oncology University of Utah Salt Lake City UT USA; ^3^ Department of Radiation Oncology Memorial Sloan‐Kettering Cancer Center New York NY USA; ^4^ Department of Pathology and Laboratory Medicine Perelman School of Medicine of the University of Pennsylvania Philadelphia PA USA; ^5^ Department of Radiation Oncology Keck School of Medicine of University of Southern California Los Angeles CA USA

**Keywords:** active surveillance, Gleason score, perineural invasion, prostate cancer, prostatectomy

## Abstract

**Background:**

The significance of perineural invasion (PNI) in prostate cancer (PC) is unclear. A recent report of patients with pT2N0R0 PC found that PNI at prostatectomy was independently associated with higher Gleason score and more diffuse prostatic disease. We aimed to test our hypothesis that PNI on prostate biopsy in pT2N0R0 patients is associated with increased Gleason score upgrading at prostatectomy.

**Methods:**

We identified 2892 patients status post prostatectomy with pT2N0R0 PC from three institutions, diagnosed between 1 January 2008 and 31 December 2014. Multivariable logistic regression (MVA) was used to evaluate the association between prostate biopsy PNI status and surgical Gleason upgrading, while controlling for potential confounders.

**Results:**

Of the 2892 patients identified, 14% had PNI on biopsy, of whom 21% had surgical Gleason upgrading, while 28% without PNI on biopsy had such upgrading (*P* < .01). On MVA, the odds ratio (OR) of surgical Gleason upgrading for patients with biopsy PNI relative to patients without biopsy PNI was 0.69 (*P* < .01). The variables associated with surgical Gleason upgrading were age ≤60 years (OR 1.22, *P* = .02) and preoperative PSA >4 ng/mL (OR 1.26, *P* = .02).

**Conclusions:**

In post‐prostatectomy patients with favorable‐risk PC, PNI on prostate biopsy was not associated with surgical Gleason score upgrading. This may be due to the association of PNI with more diffuse disease, leading to increased biopsy tumor yield and grading accuracy. These findings suggest that in this setting, biopsy PNI alone should not be a concern for more aggressive disease requiring pathologic confirmation or intervention. This may help guide treatment decision‐making for men debating active surveillance, radiation, and surgery.

## Graphical abstract

In our large, multi‐institutional report of post‐prostatectomy patients with favorable‐risk prostate cancer, biopsy perineural invasion positivity was not associated with surgical Gleason score upgrading. For patients with favorable‐risk, clinically localized prostate cancer, biopsy perineural invasion alone should not be a concern for more aggressive disease requiring pathologic conformation or intervention, and these findings may help guide treatment decision‐making.

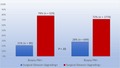

## 1 INTRODUCTION

Prostate cancer (PC) is the most common noncutaneous malignancy in American men, with an estimated 174 650 new cases in 2019.^1^ At the time of diagnosis, numerous factors, including Gleason grade, stage, prostate‐specific antigen (PSA), and comorbidities/life expectancy, are taken into account to guide potential management options.^2^ While these characteristics have well‐established relationships with PC recurrence and/or survival, the significance of perineural invasion (PNI) is less clear.^3^, ^4^, ^5^

PNI, which is infiltration of cancer cells into the perineural space, along or around a nerve,^6^ is present in roughly 22%‐65% of PC specimens in patients with organ‐confined (eg, pT2) disease.^7^ Prior analyses of PNI in PC generated conflicting results regarding the association of PNI at the time of prostatectomy with biochemical recurrence (BCR), though may have been subject to confounding due to inclusion of patients with pT2 and pT3 disease, with differing baseline BCR risks.^8^, ^9^, ^10^, ^11^, ^12^, ^13^, ^14^ A recent large multi‐institutional analysis attempted to minimize potential confounders by assessing only patients with margin‐negative (eg, R0), pT2N0M0 disease for BCR. This report found that PNI at prostatectomy was not independently associated with BCR, but was associated with higher surgical Gleason grade and greater volume of disease within the prostate using logistic regression, as compared to specimens without PNI.^7^

Given the association of surgical PNI with higher grade and volume of disease, we aimed to assess whether PNI on prostate biopsy in apparently favorable‐risk patients is associated with increased risk of Gleason score upgrading at prostatectomy in patients with pT2N0R0 disease. We hypothesized that the presence of PNI on biopsy would be associated with an increased risk of surgical Gleason score upgrading in this population.

## 2 METHODS

### 2.1 Patient population

We performed an IRB‐approved retrospective electronic medical record (EMR) review of patients from three institutions: Los Angeles County hospital (LAC), University of Southern California Norris Comprehensive Cancer Center (USC), and the Hospital of the University of Pennsylvania (Penn). LAC is a large safety‐net hospital, while USC and Penn are National Cancer Institute (NCI)‐designated comprehensive cancer centers within private university hospitals. The sources of patient data were the LAC Cancer Surveillance Program, the USC Registry (part of the NCI Surveillance, Epidemiology, and End Results cancer registry program), and the Penn Data Analytics Center. Inclusion criteria were patients with PC diagnosed between January 2008 and December 2014 treated with prostatectomy, with pT2N0R0 disease. Exclusion criteria included receipt of radiotherapy (RT) or androgen deprivation therapy (ADT) prior to prostatectomy, Gleason grade <6, unknown biopsy or surgical Gleason grade, or unknown surgical PNI status.

### 2.2 Data collection

Patients’ demographic (age at diagnosis, race/ethnicity, diagnosis year), clinicopathologic (biopsy and surgical PNI status and Gleason grade, clinical and pathological TNM staging), and laboratory data (PSA at diagnosis (defined as 6 months prior to or 3 months after diagnosis date), all postoperative PSA values through 1 January 2018) were reviewed in each institution's EMR and compiled in a centralized database. After discussion with institutional genitourinary pathologists, it was felt appropriate to classify patients whose biopsy PNI statuses were not reported as negative.

### 2.3 Data analysis/statistics

The primary objective of this study was to report and compare the rate of surgical Gleason score upgrading (from biopsy Gleason score to surgical Gleason score) based upon biopsy PNI status. A secondary objective of this study was to evaluate for associations between various patient/disease characteristics and surgical Gleason score upgrading. Another secondary objective was to compare the rate of surgical Gleason score upgrading based upon biopsy PNI status in recategorized subsets of our patient population (eg, “strict” active surveillance (AS) eligible cohort (clinical stage ≤T2a, PSA density <0.15 ng/mL, PSA <10 ng/mL, biopsy Gleason score ≤6, ≤2 positive biopsy cores, and ≤50% cancer involvement in any biopsy core)^15^ and “expanded” AS eligible cohort (any age with biopsy Gleason score ≤6 and PSA ≤10 ng/mL, or age ≥70 years with PSA ≤15 ng/mL or Gleason score ≤3+4 = 7)^16^). Such analyses will allow us to assess this relationship in more favorable or less favorable subgroups within our population.

Descriptive statistics, student's *t*, and Chi‐squared tests were used to examine differences in patient‐specific variables according to surgical Gleason upgrading status. Multivariable logistic regression (MVA) was used to evaluate the association between biopsy PNI status and surgical Gleason score upgrading, while controlling for potential confounding variables. Data were analyzed using the R programming language (*v*3.5.1, http://www.r-project.org). All *P*‐values were two‐sided, with values <.05 meeting statistical significance.

## 3 RESULTS

**Figure 1:**
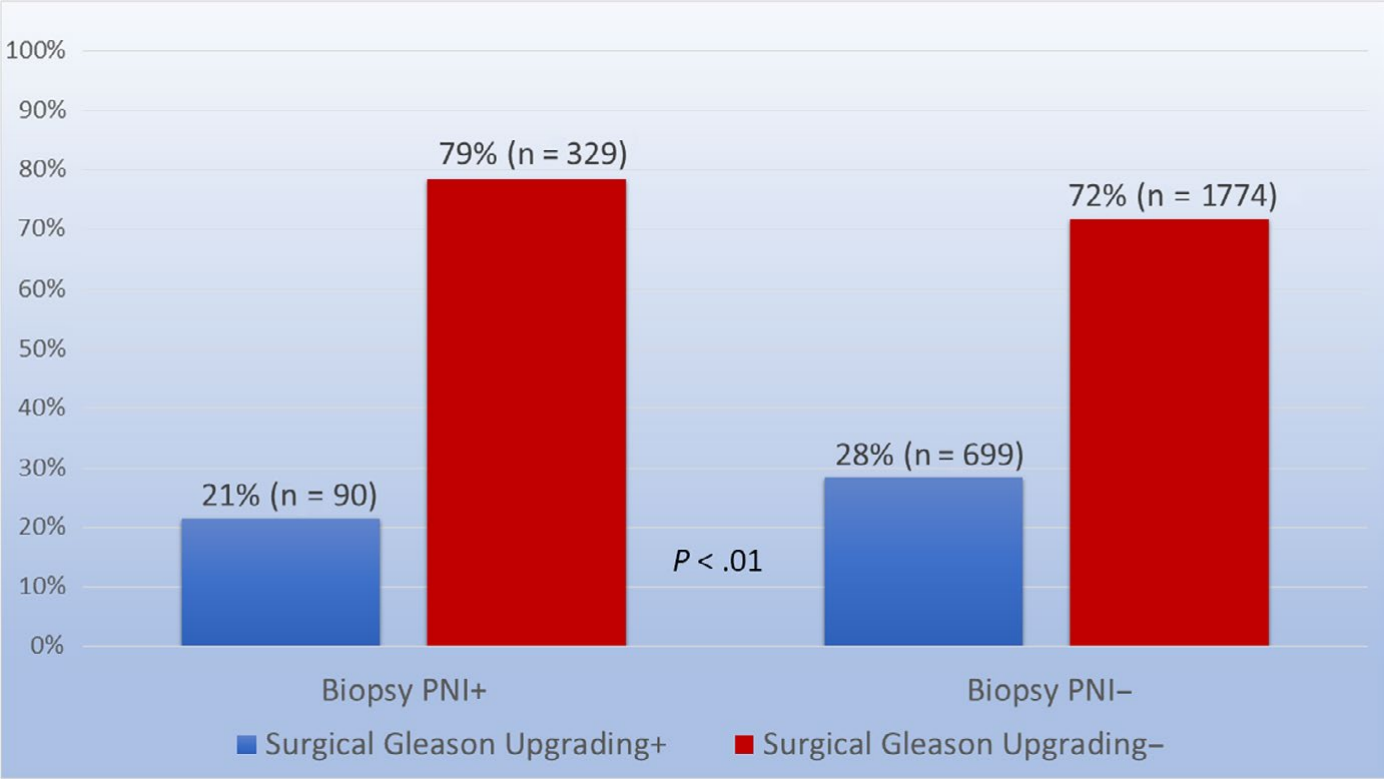
Association of biopsy PNI status with surgical Gleason upgrading. Surgical Gleason score upgrading by biopsy PNI status. Of patients with PNI on biopsy, 90 (22%) experienced surgical Gleason score upgrading. Of patients without PNI on biopsy, 699 (28%) experienced surgical Gleason score upgrading (*P* < .01)

We identified 2892 patients across all three institutions that met our inclusion criteria. Baseline patient characteristics are presented in Table 1. Within the entire cohort, 789 (27%) experienced surgical Gleason upgrading, while 2,103 (73%) did not. At baseline, patients who experienced surgical Gleason upgrading were more likely to be younger (mean 59 vs 60 years, *P* = .03), have higher pathological T stage (84% vs 80% pT2c, *P = *.02), and surgical Gleason grade (100% vs 55% Gleason 7‐10, *P* < .001), and have surgical PNI (79% vs 76%, *P* = .01) than those who did not experience surgical Gleason upgrading (Table 1). Of all patients, 419 (14%) were positive for PNI on biopsy, while 2473 (86%) were negative. Of patients with PNI on biopsy, 90 (22%) experienced surgical Gleason score upgrading. Of patients without PNI on biopsy, 699 (28%) experienced surgical Gleason score upgrading (*P* < .01) (Figure 1). Surgical Gleason grades listed by biopsy Gleason grade are shown in Table 2. On MVA, the odds ratio (OR) of surgical Gleason score upgrading for patients with PNI on biopsy relative to those without was 0.69 (95% confidence interval (CI) 0.53‐0.87, *P* < .01). Variables statistically significantly associated with surgical Gleason score upgrading on MVA were age ≤60 years (OR 1.22, 95% CI 1.03‐1.44, *P* = .02) and preoperative PSA >4 ng/mL (OR 1.26, 95% CI 1.05‐1.53, *P = *.02). When repeating the MVA including only patients with clinical T1 disease, similar associations with surgical Gleason score upgrading persisted: biopsy PNI positivity (OR 0.68, 95% CI 0.51‐0.89, *P* < .01), age ≤60 years (OR 1.22, 95% CI 1.02‐1.47, *P* = .03), and preoperative PSA >4 ng/mL (OR 1.29, 95% CI 1.01‐1.66, *P* = .045). When repeating the MVA including only patients with clinical T2 disease, there was no association of surgical Gleason upgrading with biopsy PNI positivity (OR 0.85, 95% CI 0.47‐1.53, *P* = .59).

**Table 1:** Baseline patient and disease characteristics

Patient or disease characteristic	No surgical Gleason upgrading	Surgical Gleason upgrading	*P*‐value^a^
Number of patients	2103 (73%)	789 (27%)	
Age (y)			**.03**
Mean (standard deviation)	60 (7)	59 (7)	
Race			.36
Black	302 (14%)	106 (13%)	
White	1588 (76%)	612 (78%)	
Other	213 (10%)	71 (9%)	
Treatment center			.13
LAC	37 (2%)	14 (2%)	
USC	250 (12%)	99 (13%)	
Penn	1816 (86%)	676 (86%)	
Preoperative prostate‐specific antigen (ng/mL)			.10
Mean (standard deviation)	5.8 (4.3)	6.2 (6.0)	
Clinical T Stage			.64
T1	1744 (83%)	669 (85%)	
T2	314 (15%)	107 (14%)	
T3	10 (0%)	3 (0%)	
Unknown	35 (2%)	10 (1%)	
Pathological T stage			**.02**
T2	72 (3%)	28 (4%)	
T2a	255 (12%)	61 (8%)	
T2b	90 (4%)	41 (5%)	
T2c	1686 (80%)	659 (84%)	
Surgical Gleason grade			**<.01**
5	5 (0%)	0 (0%)	
6	927 (44%)	1 (0%)	
7	1103 (52%)	719 (91%)	
8‐10	68 (3%)	68 (9%)	
Surgical perineural invasion			**.01**
Yes	1606 (76%)	623 (79%)	
No	329 (16%)	90 (11%)	
Not stated	168 (8%)	76 (10%)	

a Student's *t* and Chi‐squared tests were used for continuous and categorical variables, respectively.

**Table 2:** Surgical Gleason grades by biopsy Gleason grade

	Surgical Gleason grade					
	5	6	7	8	9	10
Biopsy Gleason grade						
5	0	1	3	0	0	0
6	5	766	716	10	2	0
7	0	152	977	28	9	0
8	0	7	106	37	19	0
9	0	2	20	15	14	1
10	0	0	0	1	1	0

The same analyses were attempted in recategorized subsets of our patient population with one fitting the “strict” AS criteria and one fitting the “expanded” AS criteria, defined in the Methods section. We identified 688 patients who met the “strict” AS criteria, and of these, none experienced surgical Gleason upgrading (*P* = .046). We identified 954 patients who met the “expanded” AS criteria, and of these, five (<1%) experienced surgical Gleason upgrading (*P* < .01). Given low event rates, MVA was not performed.

## 4 DISCUSSION

Herein, we share to our knowledge one of the largest published contemporary multi‐institutional analyses of the association between PNI on prostate biopsy and surgical Gleason upgrading in patients with pT2N0R0 disease. We found that despite the association of PNI at prostatectomy with higher Gleason grade and greater volume of prostatic disease, patients with PNI on biopsy were less likely than those without PNI on biopsy to experience surgical Gleason upgrading. Furthermore, on MVA, we found that age ≤60 years and preoperative PSA ≤4 ng/mL were associated with increased surgical Gleason upgrading.

We observed a 14% rate of prostate biopsy PNI in our cohort, which is consistent with the range of biopsy PNI positivity (7%‐33%) reported in a recent meta‐analysis of 18 studies investigating this histological finding. This is also highly consistent with the 18% rate of PNI biopsy positivity when considering only studies within the meta‐analysis with more than 70% of stage pT2 cases, which more closely resembles our cohort.^17^

We observed a 27% rate of surgical Gleason upgrading in the entire cohort, with 22% and 28% (*P* < .01) of patients with and without biopsy PNI, respectively, experiencing such upgrading. These results are comparable to results from a smaller, single‐institutional, and more varied cohort in which 154 patients with Gleason 3 + 4 = 7 PC on biopsy, who were allowed to have >pT2 disease, were assessed for Gleason upgrading at prostatectomy. The authors found a 29% rate of surgical Gleason upgrading in the entire cohort, with 26% and 29% (*P* = .70) of patients with and without biopsy PNI, respectively, experiencing such upgrading.^18^ While upgrading rates were similar, the difference in upgrading between biopsy PNI‐positive and negative cohorts did not meet significance in the referenced analysis, as it did in ours. It is possible that the substantial difference in sample size between these analyses may be contributory, as well as the facts that patients in the analysis by Flood et al were allowed to have >pT2 disease and surgical margin status was not specified, thereby introducing potential confounders that could obscure a difference. Our findings were comparable to an analysis of 109 patients, of whom 26% had biopsy PNI and 71% had pT2 disease, in which concordance between biopsy Gleason grade and Gleason grade at prostatectomy was assessed. In that analysis, the Gleason grade concordance rate among patients with biopsy PNI was greater than that of patients without biopsy PNI (46% vs 33%, *p*‐value not reported), and there were no differences in concordance rate by biopsy Gleason score for patients with biopsy PNI.^19^ Our results were also consistent with a report from Princess Margaret Hospital, in which 139 patients underwent prostate biopsy followed by radical prostatectomy, and had PNI status and Gleason grade assessed. In that report, 26% of patients had biopsy PNI, and this was found to be associated with neither Gleason upgrading (*P* = .51) nor downgrading (*P* = .208) at prostatectomy.^20^

While we hypothesized that the presence of PNI on biopsy would be associated with increased surgical Gleason upgrading given the association of PNI at prostatectomy with higher grade and volume of disease,^7^ our current findings refute our hypothesis. A potential explanation may lie within the same prior findings: if PNI is associated with greater volume of disease, the biopsy specimen may be less prone to undersampling the gland, and therefore may be more representative of the true oncologic specimen than a specimen without PNI. In a separate cohort of 313 PC patients who met AS criteria from Johns Hopkins, investigators assessed the clinicopathologic characteristics of their biopsy and prostatectomy specimens and found that cases with PNI had a higher maximum percentage of cancer on biopsy and more cases with >2 positive biopsy cores than cases without PNI.^21^ Similarly, a cohort of 845 PC patients who met the Epstein AS criteria were also assessed for the clinicopathologic characteristics of their biopsy and prostatectomy specimens, and confirmed that patients with PNI on biopsy had a greater volume of disease and rate of 2 positive cores, thus supporting a potential relationship between PNI on biopsy and representative oncologic grading.^22^

Given our findings within this favorable‐risk cohort (predominantly cT1c, pT2, PSA <10, Gleason 6‐7), a germane question pertains to how biopsy PNI may impact consideration of AS. Many of the patients analyzed had low‐risk disease, and therefore could have reasonably been managed with prostatectomy, RT, or AS, with clinical equipoise.^23^ Even with the results of the ProtecT trial, which did not specifically report PNI status, some may be swayed to recommend intervention in an otherwise low‐risk PC patient by the finding of PNI on biopsy, given associations with more aggressive disease.^24^, ^25^, ^26^ Our cohort was less confounded than such prior experiences given that we excluded patients with >pT2 disease, node‐positive disease, or positive surgical margins, and therefore excluded patients who have more aggressive disease and may already be at an increased risk of surgical Gleason score upgrading, independent of biopsy PNI status. Our large, less confounded experience found that biopsy PNI was not associated with Gleason upgrading at surgery, and therefore we argue that biopsy PNI should not deter one from recommending AS in an otherwise low‐risk patient out of concern for higher occult Gleason grade.

To further address AS, we analyzed subsets of our cohort that met the Johns Hopkins “strict” and Toronto “expanded” AS criteria. When considering the 688 patients who met “strict” AS criteria, with the limitation that number of biopsy cores and PSA density data were not available for our cohort (therefore meaning we may have been too inclusive and could overestimate an observed difference), we did not find any events of surgical Gleason upgrading, regardless of biopsy PNI status, supporting the potential safety and feasibility of AS in this population. These findings are consistent with a similar report in which 596 men who met “strict” AS criteria were assessed for biopsy PNI, and no differences in adverse pathologic features were found at prostatectomy between men with and without biopsy PNI.^27^ When considering the 954 patients who met “expanded” AS criteria, surgical Gleason upgrading remained an extremely rare event (<1%) for the entire cohort. Our results differed from a similar report in which 1197 men who met “expanded” AS criteria were assessed for biopsy PNI, and those with biopsy PNI were more likely to have Gleason upgrading at prostatectomy than those without (*P* = .01).^27^ A possible explanation for this disparity may be that in that report, men with biopsy PNI were more likely to have pT3 disease (*P* < .001), which may confound their results as compared to our cohort which was restricted to pT2 patients. As such, we maintain that biopsy PNI alone should not prohibit clinicians from offering AS in patients who meet either “strict” or “expanded” AS criteria.

While most patients in our cohort had low‐risk disease, many presented with Gleason 7 disease, and therefore would meet criteria for intermediate‐risk. In intermediate‐risk PC, standard treatment options include prostatectomy, RT, or, in select cases, AS. Given higher risk disease with a Gleason grade of 7, some may be swayed to recommend surgery over RT to have “definitive” pathological staging, particularly if PNI biopsy was positive, for the reasons previously mentioned. Still, even when excluding cT1c disease (therefore assessing only higher clinical stage patients), we did not find an increase in surgical Gleason upgrading based upon biopsy PNI positivity. Therefore, we argue that RT may be safely recommended in patients with biopsy PNI positivity, without the fear of missing higher grade disease that would only be discovered surgically. Furthermore, some institutions offer ADT with RT for PNI alone, in the absence of other factors warranting ADT, based upon data that biopsy PNI independently predicted worse clinical outcomes in patients undergoing high‐dose external beam RT, while noting that the finding was most pronounced in patients with Gleason 8‐10 disease.^28^ Our data suggest that in this apparently favorable‐risk cohort, given that patients with biopsy PNI positivity did not experience increased Gleason upgrading, the necessity of ADT for PNI alone may warrant further investigation. In addition, the MVA association we observed between young age, preoperative PSA >4, and increased Gleason upgrading at prostatectomy is thought‐provoking, and is another factor one can weigh when considering surgery, RT, or AS for PC.

Our report has several limitations. Our study design was retrospective. We did not have centralized pathology review, and tertiary Gleason grade was neither routinely reported nor considered for upgrading. PNI was reported as a binary (ie, nonquantitative) variable, and therefore assessments of biopsy PNI extent with Gleason upgrading could not be assessed. PNI biopsy status was often not reported in the reports assessed. After discussion with institutional genitourinary pathologists, it was felt appropriate to classify patients with missing biopsy PNI statuses as negative. Despite that the majority of cases, per published percentages of biopsy PNI, should be negative for this finding, it is still possible that some were consequently misclassified. Additionally, while a strength of our pT2N0R0 cohort is minimization of confounding given the associations between pT3 disease, surgical margin status, and aggression of PC, it is also possible that by excluding >pT2 patients, we may be underestimating the risk of upgrading by biopsy PNI status if it is driven by patients with extraprostatic disease, which has been associated with biopsy PNI positivity.^27^ Further study assessing the association of biopsy PNI with surgical Gleason score upgrading in an isolated, higher risk population (eg, patients with pT3‐4 disease or positive margins) would be valuable in the future.

In our large, multi‐institutional report of post‐prostatectomy patients with favorable‐risk PC, biopsy PNI positivity was not associated with surgical Gleason score upgrading. This may be due to the association of PNI with more diffuse disease, leading to increased biopsy tumor yield, and more accurate biopsy grading. For patients with favorable‐risk, clinically localized PC, biopsy PNI alone should not be a concern for more aggressive disease requiring pathologic conformation or intervention. These findings may help guide treatment decision‐making for men debating AS, RT, and surgery.

## CONFLICT OF INTEREST

The authors have no conflict of interests or disclosures relevant to this work.

## AUTHOR CONTRIBUTIONS

ARB: Conceptualization, data curation, methodology, formal analysis, project administration, writing (original draft, review, and editing); RDK: Data curation, writing (review and editing); RC: Conceptualization, methodology, formal analysis, writing (original draft, review, and editing); PMGS: Conceptualization, data curation, writing (review and editing); CL: Data curation, writing (review and editing); LES: Data curation, writing (review and editing); LKB: Conceptualization, methodology, project administration, writing (review and editing); NV: Conceptualization, methodology, project administration, writing (review and editing).

## Data Availability

The data that support the findings of this study are available from the corresponding author upon reasonable request.
